# ID 200 - Avaliação da Razão Custo-Utilidade Incremental da Terapia por Pressão Negativa em Pacientes com Lesões Complexas de Pé Diabético Comparada à Terapia com Curativo Tradicional: uma análise para o contexto brasileiro

**DOI:** 10.5327/2237-9622.2025.v34s1.200

**Published:** 2025-11-25

**Authors:** Cecília de Oliveira Carvalho Faria, Bertha Lúcia Costa Borges, Fábio França Silva, Klébya Hellen Dantas de Oliveira, Lucia Reis Nascimento

**Keywords:** terapia por pressão negativa, pé diabético, custo-utilidade, modelagem econômica, políticas públicas

## Abstract

**Introdução:**

O pé diabético é uma das complicações mais graves do diabetes mellitus (DM), levando a custos elevados tanto para o paciente quanto para o sistema de saúde. Estratégias de tratamento que acelerem a cicatrização e reduzam o número de amputações são necessárias. A terapia por pressão negativa (TPN) tem mostrado resultados promissores em termos de eficácia, com maior taxa de cicatrização e menor tempo até a recuperação, mas sua viabilidade econômica permanece incerta no contexto brasileiro.

**Método:**

Este estudo utilizou um modelo econômico de custo-efetividade para comparar a TPN com a terapia com curativo tradicional (TCT) em pacientes com lesões complexas de pé diabético. A árvore de decisão foi construída com dados de ensaios clínicos randomizados, avaliando os estados de amputação e cicatrização ao longo de um ciclo anual. A perspectiva adotada foi a do Sistema Único de Saúde (SUS), considerando os custos de 2023. Análises de sensibilidade determinística e probabilística foram conduzidas para avaliar a incerteza no modelo, utilizando o software TreeAge Pro.

**Resultados:**

A TPN apresentou maior efetividade (0,79 AVAQ verus 0,72 AVAQ para a TCT), porém com um custo mais elevado (R$ 9.184,27 vs. R$ 3.961,45), resultando em um custo incremental de R$ 5.222,82. A razão de custo-efetividade incremental (RCUI) foi de R$ 72.035,87 por ano de vida ajustado pela qualidade (QALY). A análise de sensibilidade determinística indicou que o da cicatrização e o custo do curativo simples são os principais fatores que afetam a RCUI. Já a análise probabilística demonstrou que, em mais de 50% das simulações, a TPN situou-se acima do limiar de custo-efetividade brasileiro de R$ 40.000,00 por QALY.

**Conclusão:**

Embora a TPN tenha mostrado maior efetividade, o custo incremental é significativo, superando o limiar estabelecido para o contexto brasileiro. A análise de sensibilidade indica que as utilidades relacionadas à cicatrização e o tipo de curativo utilizado no TCT têm grande impacto nos resultados, sugerindo que a adoção de coberturas especiais na TCT pode melhorar sua relação custo-efetividade. A TPN não se configurou como uma estratégia dominante, pois apresentou custos mais elevados para ganhos modestos em efetividade. No entanto, apesar de não reduzir diretamente a expectativa de vida, a terapia pode proporcionar melhor qualidade de vida ao evitar complicações, como infecções e amputações, que são associadas a períodos sem ulceração.

Para que a TPN se torne custo-efetiva no Brasil, seria necessário ajustar o limiar de custo-efetividade para cerca de 1,5 a 2 vezes o PIB per capita e negociar melhores preços no fornecimento da tecnologia. A implementação dessa estratégia pode ser justificada se acompanhada de parcerias que busquem reduzir os custos da TPN, o que tornaria seu uso mais sustentável no SUS. Esses resultados podem apoiar tomadores de decisão na seleção de tratamentos mais adequados para o paciente portador do pé diabético, levando em consideração a escassez de recursos e a alta carga econômica associada a essa complicação.

